# Psychological empowerment and relational catalysis: a quasi-experimental study on graduate education evaluation reform in the AI era

**DOI:** 10.3389/fpsyg.2026.1897845

**Published:** 2026-08-12

**Authors:** Kefei Tan, Bing Gao

**Affiliations:** Department of Educational, Minzu University of China, Beijing, China

**Keywords:** artificial intelligence, evaluation reform, graduate education, humble supervisor leadership, psychological empowerment

## Abstract

**Introduction:**

The rise of generative AI (e.g., ChatGPT, DeepSeek) challenges traditional graduate evaluation systems centered on individual theses and standardized examinations. Current institutional responses—task redesign and AI skills training—have shown limited effectiveness, as they overlook the psychological internalization process, teacher-student relational dynamics, and their synergistic effects.

**Methods:**

This study conducted a three-arm quasi-experiment with 155 doctoral students in education over one semester. Group A (traditional model) received no AI guidance; Group B received AI skills training only; Group C received comprehensive reform combining AI training, practical team-based projects, and humble mentor leadership training. Psychological empowerment was measured as a mediator, and humble mentor leadership as a moderator. Data were analyzed using regression with cluster-robust standard errors and bootstrap tests.

**Results:**

Group C scored significantly higher than Groups A and B on course satisfaction, professional competence, and psychological empowerment (all *p* < 0.001). Psychological empowerment showed a significant indirect association with learning outcomes (indirect effect = 0.22, 95% CI [0.12, 0.34]), with meaning and autonomy as the core dimensions. Humble mentor leadership significantly moderated the engagement–empowerment path within Group C (*β* = 0.24, *p* < 0.001), with the conditional indirect effect significant only under high humble leadership.

**Discussion:**

These findings suggest that the synergy of practical task redesign, psychological empowerment, and humble mentor leadership may be associated with positive student outcomes in AI-era educational reform. However, due to the cluster-assigned design with one cluster per condition and non-equivalent assessment tasks across groups, all results should be interpreted as exploratory and hypothesis-generating rather than causal. Future research should employ multi-cluster or individually randomized designs with common assessment tasks to permit valid causal inference.

## 1 Introduction

Generative artificial intelligence is rapidly embedding itself into the core of higher education. When models such as ChatGPT and DeepSeek can complete tasks like outlining a graduate thesis, conducting literature reviews, and even solving complex problems, the traditional graduate academic evaluation system—centered on “individual thesis + standardized examination”—faces a fundamental question: when knowledge output is no longer exclusively human, what are we actually evaluating (Zhai et al., 2023; Zheng et al., 2024)? This issue has pushed the impact of AI on higher education beyond technical operational concerns to the fundamental pedagogical questions of why we learn, how we teach, and how we evaluate—these underlying logics are being re-examined (Ng et al., 2023).

This fundamental challenge has prompted institutions to seek adaptive responses. Current institutional responses can be summarized as two main paths. The first is a technology-defense path—designing task formats that AI cannot directly replace, such as project-based learning and oral defenses, to maintain the “AI-resistance” of evaluation (Kasneci et al., 2023; Mollick and Mollick, 2023). The second is a skills-empowerment path—providing students with systematic training in AI tool usage to enable them to harness AI rather than be replaced by it (Lu et al., 2024). While both paths have shown some effectiveness in practice, emerging evidence suggests that task complexity alone does not guarantee intrinsic engagement, and skill mastery alone does not automatically translate into competence transformation (Wang et al., 2023; Liu and Zhang, 2024). More critically, both paths tend to overlook three critical gaps: (1) the psychological process of learner internalization, (2) the role of teacher-student relational dynamics, and (3) the synergies of combining task redesign, psychological empowerment, and humble leadership.

The above critiques clearly reveal the shortcomings of existing approaches, but alternative mechanisms still lack sufficient empirical testing. If a reform scheme that goes beyond “prevention” and “training” exists, what should it consist of? What are its boundary conditions? This study attempts to answer these questions. At the task level, reform needs to embed practical, authentic tasks on top of skills training; at the psychological level, these external interventions need to be transformed into a sense of meaning and autonomy—core dimensions of psychological empowerment (Spreitzer, 1995; Zhang and Bartol, 2010); at the relational level, humble mentor leadership (Owens et al., 2013) needs to be introduced as a “relational catalyst” for fully activating psychological empowerment. Psychological empowerment has been shown to be a key antecedent of students’ deep learning and innovative behavior (Li et al., 2006), and humble leadership—characterized by openness, self-reflection, and teachability—can create a psychologically safe relational environment that amplifies the internalization of external interventions (Hu et al., 2022).

Based on Self-Determination Theory (Ryan and Deci, 2000) and Leader-Member Exchange Theory (Graen and Uhl-Bien, 1995), this study constructed a moderated mediation model: psychological empowerment as the mediator and humble mentor leadership as the moderator. It should be noted in advance that, because the research procedure provided an additional Humble Leadership Practice Manual to mentors in Group C, while mentors in Groups A and B only received a neutral workshop, the moderation analysis of humble leadership was limited to within Group C (using the individual-level “depth of engagement with practical tasks” as the independent variable, see Section 2.4), so as to avoid construct validity problems arising from confounding intervention components. Cross-group differences in humble leadership are reported only descriptively and are not used for causal inference.

This study draws on two complementary theories to explain how and under what conditions AI-era evaluation reform influences learning outcomes.

First, Self-Determination Theory (SDT; Ryan and Deci, 2000) posits that intrinsic motivation and deep learning arise when three basic psychological needs—autonomy, competence, and relatedness—are satisfied. In educational settings, psychological empowerment (Spreitzer, 1995) operationalizes these needs through four dimensions: meaning (the alignment of tasks with personal values, akin to relatedness and purpose), competence (self-efficacy), autonomy (self-direction), and impact (the sense of making a difference). We therefore propose psychological empowerment as a statistical bridge through which external reform (task design) influences learning outcomes. SDT suggests that simply providing AI tools or complex tasks is insufficient; students must perceive these activities as meaningful and self-directed to internalize the learning process.

Second, Leader-Member Exchange (LMX) theory (Graen and Uhl-Bien, 1995) explains how the quality of the relationship between supervisors and subordinates shapes the observed patterns associated with interventions. In the AI-era classroom, humble mentor leadership—characterized by openness, self-reflection, and teachability (Owens et al., 2013)—represents a specific form of high-quality LMX. Unlike traditional authoritative supervision, humble leadership fosters psychological safety (Edmondson, 1999), which is particularly important when tasks are novel, uncertain, and collaborative. We argue that humble leadership acts as a relational catalyst that strengthens the translation of challenging practical tasks into psychological empowerment. This moderating role is expected to be context-dependent: it should emerge only when tasks require exploration and risk-taking (as in Group C), not when tasks are routine and individual (as in Groups A and B).

**Figure 1:**
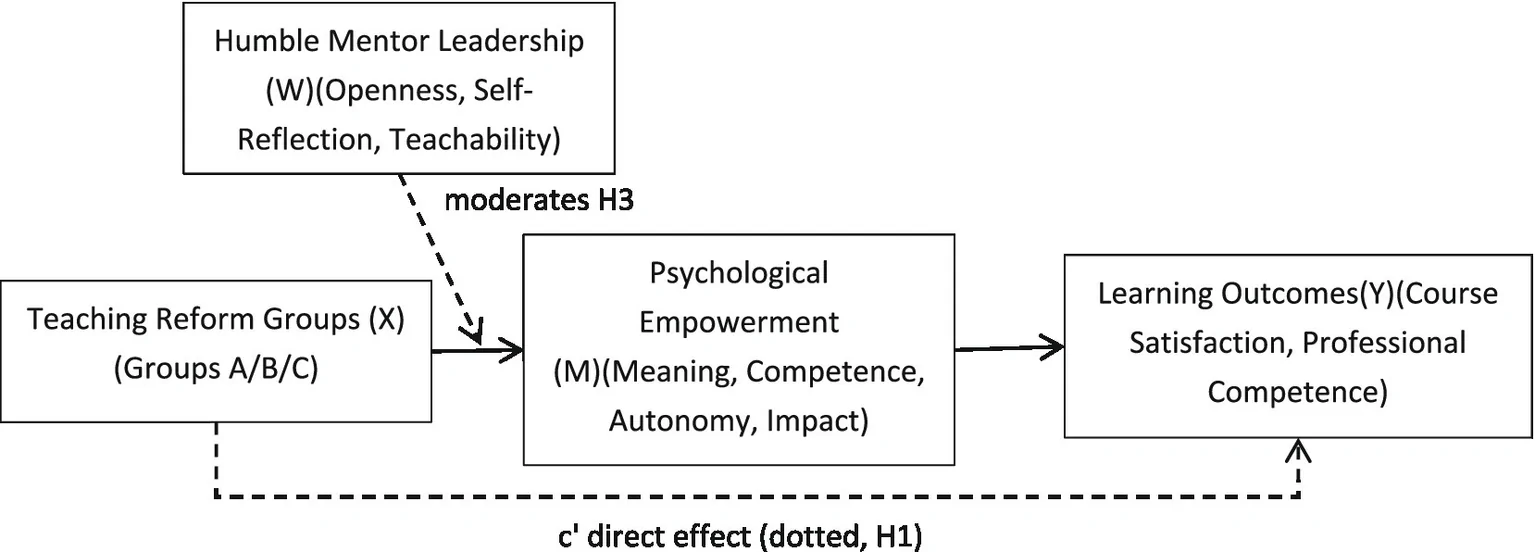
Theoretical framework. W moderates the X → M relationship (first-stage moderation). The dotted line represents the direct effect. The moderating effect was examined only within Group C using individual-level engagement depth as X, while the main effect of the reform group (H1) was tested using the three-group categorical variable.

Integrating SDT and LMX, the present study constructs a moderated mediation model (Figure 1): psychological empowerment is proposed as a statistical bridging mechanism between the reform and learning outcomes (H2), and humble mentor leadership moderates the path from reform engagement to psychological empowerment (H3). This framework moves beyond single-path explanations by specifying both the internal psychological mechanism and the relational boundary condition.

The study conducted a three-arm quasi-experiment with doctoral students in education, testing the following hypotheses:

*H1* (main effect): The comprehensive reform group (Group C) is expected to show higher scores in learning outcomes (course satisfaction and professional competence) than the AI training group (Group B) and the traditional model group (Group A); Group B is also expected to show higher scores than Group A. Given the cluster-assigned design, this hypothesis is tested as an exploratory comparison rather than a confirmatory test.

*H2* (indirect effect): Psychological empowerment (especially the meaning and autonomy dimensions) would play a statistical indirect role between the reform and learning outcomes.

*H3* (within-treatment interaction, within Group C): Within Group C, perceived humble mentor leadership (measured at T2, reflecting a combination of natural differences in supervisor behavior and experimental induction via the practice manual) positively moderates the path from individual-level engagement depth (X_individual) to psychological empowerment (M). The statistical indirect association between engagement and learning outcomes via psychological empowerment is expected to be stronger for students who perceive higher levels of humble leadership from their supervisors. Crucially, this hypothesis tests a within-treatment interaction and does not purport to test humble leadership as an independent, naturally occurring moderator across all conditions.

## 2 Research design

### 2.1 Experimental design

A three-arm pre-test/post-test cluster-assigned quasi-experiment was adopted. A quasi-experimental design was chosen because individual random assignment was not feasible given the existing course structure; one existing cohort was assigned to each condition. As a result, condition is potentially confounded with cohort and intake timing. Cluster-level assignment preserved ecological validity while allowing comparisons across conditions. However, we acknowledge that this design cannot achieve the same level of causal inference as an individually randomized controlled trial. Therefore, we interpret the results as suggestive of effects rather than as definitive causal evidence, and between-group differences should be read with appropriate caution.

The independent variable was teaching reform group (Group A: traditional thesis model; Group B: AI training + traditional tasks; Group C: AI training + practical task-based evaluation reform). The mediator was psychological empowerment (meaning, competence, autonomy, impact). The dependent variables were learning outcomes (course satisfaction, professional competence). The moderator was humble mentor leadership (openness, self-reflection, teachability), whose moderating effect was tested only within Group C (using individual-difference variables within Group C). The study tested the main effects, indirect effects, and within-Group C moderating effect while controlling for T1 psychological empowerment, AI familiarity, self-rated academic foundation, cooperative tendency, supervisor guidance time, and demographic variables.

A critical limitation of this design bears repeating: with only one cluster per condition, the observed between-group differences may reflect not only the interventions but also pre-existing differences between cohorts, intake timing, or other unmeasured cluster-level factors. Therefore, we frame this study as a preliminary investigation intended to generate hypotheses for future research, rather than as a definitive test of causal effects. A further critical caveat is warranted regarding the statistical implications of this design. With only one cluster per condition, the effective sample size for testing between-group treatment effects is essentially three clusters rather than 155 individual participants. Under these circumstances, cluster-robust standard errors are unlikely to provide reliable inference, and the reported *p*-values and confidence intervals should be interpreted as descriptive and exploratory rather than confirmatory.

*Statistical software*: All analyses were conducted using SPSS 28.0 (IBM Corp.) with the PROCESS macro version 4.2 (Hayes, 2022) for moderated mediation. Cluster-robust standard errors for between-group comparisons were computed using the sandwich package (Zeileis, 2004) in R version 4.2.2.

### 2.2 Participants and group assignment

Participants were drawn from three parallel cohorts of full-time doctoral students in education at two “Double First-Class” universities in Beijing and Shanghai: one cohort from the fall 2025 intake and two cohorts from the spring 2026 intake. Pre-test at the beginning of the semester showed no significant differences among the three groups in demographic variables, AI tool familiarity, initial psychological empowerment level, academic foundation, cooperative tendency, or expected supervisor guidance time (*p* > 0.05), indicating acceptable initial equivalence after cluster assignment (see Table 1). Of note, this is a cluster-randomized design (classrooms as clusters, *k* = 3) rather than individual randomization. Accordingly, we interpret between-group differences with appropriate caution. Cluster random assignment was used: each cohort was randomly assigned to one of the three conditions. The final sample distribution was: Group A (control, *n* = 52), Group B (AI training, *n* = 51), Group C (comprehensive reform, *n* = 52), total *N* = 155.

**Table 1:** Descriptive statistics and difference tests for pre-test variables across the three groups.

Variable	Group A (*n* = 52)	Group B (*n* = 51)	Group C (*n* = 52)	F	*p*
T1 psychological empowerment	4.01 ± 0.80	3.98 ± 0.81	4.03 ± 0.79	0.14	0.869
AI familiarity	2.40 ± 1.10	2.38 ± 1.12	2.42 ± 1.09	0.08	0.923
Self-rated academic foundation	3.88 ± 0.96	3.85 ± 0.97	3.91 ± 0.95	0.11	0.896
Cooperative tendency	3.80 ± 0.80	3.82 ± 0.79	3.81 ± 0.81	0.04	0.961
Gender (male/female)	29/23	25/26	26/26	—	0.952
Age (years)	32.6 ± 4.3	32.9 ± 4.4	32.7 ± 4.2	0.15	0.861
Work experience (years)	8.7 ± 3.6	8.8 ± 3.7	8.7 ± 3.5	0.06	0.942

*Sample size justification*: For a medium-sized statistical indirect association (*α* = 0.05, power = 0.80), Fritz and MacKinnon (2007) recommended at least 70 participants per group. With approximately 52 participants per group, the present study is adequately powered to detect large effects but may have limited power for smaller effects.

*Post-hoc* power analysis: Although our *a priori* sample size estimation was based on detecting a medium-sized statistical indirect association, we also conducted *post-hoc* power analyses for the key tests. For the interaction effect in Group C (*β* = 0.24, *n* = 52), using G*Power 3.1 for a multiple regression with two predictors and an interaction term (f^2^ = 0.09, based on the observed *R*^2^ change of 0.03), the achieved power was 0.71. For the index of moderated mediation (observed effect size = 0.09), Monte Carlo simulation (5,000 draws) with the observed parameter estimates yielded empirical power of 0.79. These values are close to or slightly below the conventional threshold of 0.80. We acknowledge that power for detecting smaller effects would be insufficient, and null findings (e.g., the non-significant interaction in Groups A and B) should be interpreted with caution.

*Clustering structure description*: This study used cluster assignment (classrooms as clusters, *k* = 3) rather than individual random assignment. Due to the small number of clusters, stable multilevel modeling was not feasible, and the three groups are potentially confounded with cohort and intake timing. To conservatively estimate standard errors, all regression analyses involving between-group comparisons used cluster-robust standard errors (clustering at the classroom/supervisor level). The intraclass correlation coefficients (ICCs) for the main outcomes were: course satisfaction ICC = 0.12, professional competence ICC = 0.09, psychological empowerment ICC = 0.11, indicating some degree of within-class similarity but not a strong clustering level (usually ICC > 0.20 is considered strong clustering). *Post-hoc* comparisons used Tukey’s HSD correction for multiple comparisons. Limitations of this approach are addressed in the Discussion.

### 2.3 Instruments and measures

All measures used a 6-point Likert scale (1 = strongly disagree, 6 = strongly agree) and were administered anonymously online at the beginning (T1) and end (T2) of the semester.

#### 2.3.1 Psychological empowerment scale

The Chinese version (Li et al., 2006) of Spreitzer’s (1995) scale was used, with 12 items covering four dimensions: meaning (3 items, e.g., “The learning tasks I participate in align with my personal values”), competence (3 items, e.g., “I have the abilities needed to complete my learning tasks”), autonomy (3 items, e.g., “I can decide on my own how to complete my tasks”), and impact (3 items, e.g., “My learning outcomes can influence the resolution of relevant issues”). It should be noted that in Spreitzer’s (1995) original organizational context, “impact” refers to an individual‘s influence on work-unit outcomes—the degree to which one can affect strategic, administrative, or operating outcomes. In adapting this dimension to doctoral education, we operationalized impact as the extent to which a student’s academic views, research proposals, or problem-solving contributions influence team decisions, group collaboration, or practical project outcomes. This respecification maintains the core meaning of “making a difference” while shifting the unit of analysis from organizational work units to collaborative learning teams. The four-factor CFA (reported above) supports the discriminant validity of this dimension in the educational context. Cronbach’s *α* was 0.93 at T1 and 0.94 at T2; subscale α ranged from 0.86 to 0.91.

*Confirmatory factor analysis (CFA)*: A four-factor CFA on T2 data (*N* = 155) showed good model fit: *χ*^2^/df = 2.31, CFI = 0.94, TLI = 0.92, RMSEA = 0.07, SRMR = 0.05. The average variance extracted (AVE) for each dimension was >0.50, and the square root of AVE was larger than the inter-dimension correlations, indicating acceptable discriminant validity.

*Common method bias test*: Harman’s single-factor test extracted four factors with eigenvalues >1 from unrotated exploratory factor analysis, with the first factor explaining 28.6% of the total variance (below the 40% threshold). In addition, the unmeasured latent method factor approach showed no significant improvement in model fit after adding a method factor (ΔCFI<0.01), suggesting that common method bias was not severe. To further rule out common method bias, we conducted a marker variable analysis (Lindell and Whitney, 2001) using a theoretically unrelated variable (“preference for coffee”) that was measured at T2 with a single item (“I prefer coffee over tea,” 1 = strongly disagree, 6 = strongly agree). This item was chosen because it has no theoretical overlap with the main constructs under investigation, serving as a proxy for method effects unrelated to the substantive variables. The correlations among the main study variables remained significant after partialling out the marker variable, and the pattern of significance for the indirect effects did not change. This additional test suggests that common method bias is unlikely to threaten the validity of the conclusions.

#### 2.3.2 Humble Mentor leadership scale

Based on Owens et al. (2013), the scale was adapted to the educational context as theEducational Context Humble Mentor Leadership Scale. After translation, back-translation, and a pilot test (*n* = 30), three items were adjusted. The formal scale had nine items covering three dimensions: openness (3 items, e.g., “My supervisor appreciates and learns from the novel ideas proposed by students”), self-reflection (3 items, e.g., “My supervisor openly admits his/her limitations in applying AI”), and teachability (3 items, e.g., “My supervisor proactively asks students about unfamiliar AI tools”). Administered at T2, the overall *α* was 0.91; subscale α ranged from 0.85 to 0.89. CFA showed good fit for the three-factor model (χ^2^/df = 2.45, CFI = 0.93, TLI = 0.91, RMSEA = 0.08).

Because only supervisors in Group C received the Humble Leadership Practice Manual, the variation in perceived humble leadership within Group C reflects a combination of (a) natural differences in supervisors’ baseline humility, and (b) experimental induction from the manual. Therefore, the moderating effect reported in H3 (within Group C) should not be generalized to naturally occurring humble leadership without further validation.

#### 2.3.3 Course satisfaction scale

A self-developed 6-item scale was used (e.g., “The evaluation methods of this course truly reflect what I have learned”). Confirmatory factor analysis (CFA) on the 6 items showed good fit for a one-factor model: χ^2^/df = 2.02, CFI = 0.96, TLI = 0.94, RMSEA = 0.07, SRMR = 0.04. Factor loadings ranged from 0.71 to 0.88, all significant. Composite reliability (CR) was 0.92, and average variance extracted (AVE) was 0.66. Cronbach‘s *α* was 0.92. While this scale is self-developed, the psychometric properties in this sample are acceptable.

#### 2.3.4 Professional competence assessment rubric (performance-based)

This is a performance-based assessment scored by external experts, not a self-report scale. To avoid scoring bias due to different task formats, a unified end-of-term competence assessment protocol was developed. *Assessment materials*: Groups A and B submitted individual course papers (Group B additionally provided an AI usage statement); Group C submitted a personal in-depth reflection and analysis report based on group projects (removing collaborative processes to focus on individually internalized competence). *Assessment method*: Five external experts blind to group assignment (from three different universities’ education departments) rated the materials using theCore Competence Assessment Scale, which included academic rigor and analytical depth (35 points), practical insight and innovativeness (35 points), and technology integration and critical reflection (30 points), total 100 points. Inter-rater reliability ICC(3,k) = 0.89. To ensure scoring comparability despite the different output formats, the assessment rubric was designed to focus on competence dimensions (academic rigor, analytical depth, practical insight, and critical reflection) rather than task-specific features. All expert raters were blind to group assignment. Nevertheless, the format differences between individual papers and group project reports remain a potential source of bias, and the results should be interpreted with this limitation in mind. Furthermore, because the assessment rubric included dimensions such as “technology integration” and “critical reflection”—which align closely with the specific objectives of the AI-enhanced intervention—the outcome measure may partially capture successful engagement with the intervention itself rather than underlying professional competence. This creates a degree of circularity between the intervention design and the assessment criteria. Consequently, the observed superiority of Group C should be interpreted with particular caution, as it may reflect, in part, the greater alignment between the intervention and the outcome measure rather than a genuine improvement in competence.

To mitigate concerns about potential circularity between the intervention objectives and the assessment rubric, the scoring protocol incorporated several design safeguards. First, the rubric’s core dimensions—academic rigor and analytical depth (35 points) and practical insight and innovativeness (35 points)—were designed to assess general academic competencies rather than mastery of specific AI tools. Second, the technology integration dimension (30 points) was operationalized broadly to evaluate students’ critical reflection on AI’s affordances and limitations, rather than technical proficiency with specific tools. Third, all raters were blind to group assignment. Even with these design safeguards, systematic alignment between Group C’s project task and technology-focused scoring dimensions cannot be fully eliminated.

#### 2.3.5 Control variables

The following variables were measured and controlled: (1) T1 psychological empowerment (administered at the beginning of the semester); (2) AI familiarity (single item: “Frequency of using generative AI tools,” 1 = never, 6 = daily); (3) self-rated academic foundation (single item: “Your assessment of your own foundation in education discipline theory,” 1 = very weak, 6 = very solid); (4) cooperative tendency (5 items, *α* = 0.83); (5) supervisor guidance time (measured at the end of the semester: “Time spent in individual guidance with your supervisor this semester,” 1 = <5 h, 6= > 30 h); (6) demographic variables (gender, age, years of work experience).

#### 2.3.6 Within-group C independent variable (for H3)

To test the moderating effect within Group C (H3), an additional measure of “depth of engagement with practical tasks” was administered to students in Group C. This self-developed scale had 3 items (*α* = 0.87), e.g., “I invested a great deal of time in the group project,” “I actively explored multiple AI tools in the project,” “My contribution to the project exceeded the basic requirements.” All Group C students reported this at T2. This variable captured individual differences in actual engagement under the uniform intervention condition and served as the independent variable in the H3 model.

### 2.4 Procedure and intervention

The study lasted one semester (16 weeks). After the pre-test, the intervention was implemented during weeks 2–9.

*Group A (control)*: Traditional instruction + individual paper assignments; no guidance on AI use.

*Groups B and C (AI training)*: Both received a 16-h “Critical AI Empowerment Workshop” covering prompt engineering (4 h), research ethics (4 h), data analysis (4 h), and content critique (4 h), delivered as 2 h per week for 8 weeks.

*Additional intervention for Group C (practical evaluation reform)*: 70% of the course grade was based on process-oriented and competence-based indicators (problem definition value 15%, critical AI application 20%, team collaboration and iteration 20%, presentation persuasiveness 15%), and 30% on the completeness of the project proposal. Students worked in teams (4–5 persons per group) on real educational problems (e.g., “Investigation of high-quality and balanced development of county-level compulsory education,” “Ethical risks and avoidance strategies of AI in classroom teaching”), chose their own topics, and designed research proposals.

*Supervisor variable control*: The same teaching team (8 supervisors) taught all three groups, each supervising 31–32 students. At the beginning of the semester, all supervisors attended a 3-h neutral workshop on “Teacher-student co-learning in the AI era.”Supervisors in Group C received an additionalHumble Leadership Practice Manual(containing a list of 10 specific behaviors, e.g., “Engage in co-learning of new AI functions with students at least once a week,” “Openly admit one’s own cognitive limitations during team discussions,” “Provide targeted praise for students’ new proposals”), and were asked to record their practice weekly, with a mid-term feedback session in week 8.

*A special note*: Because only Group C supervisors received the humble leadership practice manual, the source of variation in humble mentor leadership differs across the three groups. Therefore, the moderating effect analysis (H3) was limited to within Group C, using the individual-level “depth of engagement with practical tasks” as the independent variable, to avoid construct confounding in cross-group comparisons.

## 3 Results

Before presenting the results, a crucial interpretative caveat is warranted. The test of the moderating effect of humble leadership (H3) was conducted exclusively within Group C, using individual-level engagement depth as the independent variable. Because Group C was the only condition that received the Humble Leadership Practice Manual, the variation in perceived humble leadership within this group reflects a combination of natural differences in supervisor behavior and the experimental induction from the manual. Therefore, the results for H3 should be interpreted as a within-treatment interaction effect rather than as a test of a general, independent contextual moderator. The “triple synergy” framework is thus presented as a model consistent with the observed data patterns, rather than as a hypothesis that was strongly tested by the current design.

### 3.1 Between-group differences (H1)

Given that Group C differed from the other groups on multiple components, the following comparisons should be interpreted as reflecting the association with a comprehensive reform package rather than any single component. A one-way ANOVA (using cluster-robust standard errors and Tukey HSD *post-hoc* correction) showed significant differences among the three groups on post-test variables (see Table 2). Course satisfaction: Group C (*M* = 4.90, SD = 0.70) scored significantly higher than Group B (*M* = 4.35, SD = 0.75, *p* < 0.001) and Group A (*M* = 3.92, SD = 0.81, *p* < 0.001); Group B outperformed Group A (*p* < 0.01). Professional competence: Group C (*M* = 86.10, SD = 6.40) scored significantly higher than Group B (*M* = 78.80, SD = 7.00, *p* < 0.001) and Group A (*M* = 74.00, SD = 8.20, *p* < 0.001); Group B also scored significantly higher than Group A (*p* < 0.01). It is important to note, however, that the assessment materials differed between groups (group project-based reports for Group C vs. individual papers for Groups A and B). This difference in task format may have influenced expert ratings, potentially overestimating or underestimating the true competence advantage of Group C. Therefore, the observed competence scores should be interpreted with caution.

**Table 2:** Descriptive statistics and difference tests for post-test variables across the three groups (cluster-robust SE + Tukey HSD).

Variable	Group A (*n* = 52)	Group B (*n* = 51)	Group C (*n* = 52)	*F*	*p*	*Post-hoc*
Course satisfaction	3.92 ± 0.81	4.35 ± 0.75	4.90 ± 0.70	36.52	<0.001	C > B > A
Professional competence	74.00 ± 8.20	78.80 ± 7.00	86.10 ± 6.40	54.38	<0.001	C > B > A
Psychological empowerment	4.04 ± 0.80	4.27 ± 0.71	4.72 ± 0.67	25.61	<0.001	C > B > A
Meaning	4.00 ± 0.84	4.34 ± 0.78	4.95 ± 0.72	34.47	<0.001	C > B > A
Competence	4.10 ± 0.82	4.25 ± 0.76	4.65 ± 0.69	16.77	<0.001	C > B > A
Autonomy	3.97 ± 0.85	4.30 ± 0.79	4.88 ± 0.73	31.58	<0.001	C > B > A
Impact	4.05 ± 0.83	4.20 ± 0.77	4.45 ± 0.71	11.39	<0.001	C > B > A
Humble leadership	3.88 ± 0.88	3.94 ± 0.84	4.61 ± 0.75	19.85	<0.001	C > B ≈ A

Psychological empowerment: Group C (*M* = 4.72, SD = 0.67) scored significantly higher than Group B (*M* = 4.27, SD = 0.71, *p* < 0.001) and Group A (*M* = 4.04, SD = 0.80, *p* < 0.001); Group B scored significantly higher than Group A (*p* < 0.05). Among the subdimensions, meaning (*F* = 30.12, *p* < 0.001) and autonomy (*F* = 28.45, *p* < 0.001) showed the largest between-group differences. Effect sizes: partial η^2^ for group on course satisfaction = 0.22 (large effect), on professional competence = 0.30 (large effect), on psychological empowerment = 0.17 (medium to large effect).

*Missing data handling*: The T2 questionnaire return rate was 96.5% (149 of 155 participants completed all measures). Missing data were mainly from skipped individual items (missing rate <3%), and were handled using multiple imputation (5 imputations). The pattern of results did not change after imputation.

### 3.2 Statistical indirect association of psychological empowerment (H2)

This section tests H2. Because both psychological empowerment (M) and learning outcomes (Y) were measured at T2, the temporal order cannot be established. Accordingly, the following results are interpreted as statistical indirect associations in cross-sectional data (Maxwell and Cole, 2007), rather than causal mediation effects. Readers are reminded that these results are associational and do not support claims about temporal or causal sequencing.

Dummy coding. For the regression analyses, Group A (traditional model) was set as the reference category. Group B (AI training only) and Group C (comprehensive reform) were dummy-coded as 0/1 indicators. All models controlled for T1 psychological empowerment, AI familiarity, self-rated academic foundation, cooperative tendency, supervisor guidance time, gender, age, and work experience. After controlling for T1 psychological empowerment, AI familiarity, self-rated academic foundation, cooperative tendency, supervisor guidance time, and demographic variables, regression analyses (with cluster-robust standard errors) were conducted. Compared to Group A, Group C significantly predicted T2 psychological empowerment (*β* = 0.41, *p* < 0.001), and Group B also showed a significant but weaker effect (β = 0.18, *p* < 0.01). Psychological empowerment showed a strong positive association with course satisfaction (*β* = 0.53, *p* < 0.001) and professional competence (*β* = 0.49, *p* < 0.001). When the mediator was entered simultaneously with the group variable, the direct effects of group on both outcomes were non-significant (Group C → satisfaction: *β* = 0.06, *p* > 0.05; Group C → competence: *β* = 0.05, *p* > 0.05). Bootstrap tests (5,000 resamples) showed significant indirect effects of psychological empowerment: the indirect effect on course satisfaction was 0.22 (95% CI [0.12, 0.34]), and on professional competence was 0.20 (95% CI [0.10, 0.31]). This pattern was consistent with a complete statistical indirect association.

Further analyses examined the indirect effects of each dimension (see Table 3). Meaning showed the largest indirect effects on course satisfaction (indirect effect = 0.12, 95% CI [0.06, 0.20]) and professional competence (0.11, 95% CI [0.05, 0.18]), contributing more than 50% of the indirect effect; autonomy was second; competence and impact contributed less (both below 25%). These results support H2 and indicate that meaning and autonomy are the core dimensions.

**Table 3:** Indirect effects of each dimension of psychological empowerment.

Dimension	Dependent variable	Indirect effect	95% CI
Meaning	Course satisfaction	0.12	[0.06, 0.20]
Meaning	Professional competence	0.11	[0.05, 0.18]
Competence	Course satisfaction	0.05	[0.02, 0.09]
Competence	Professional competence	0.05	[0.02, 0.08]
Autonomy	Course satisfaction	0.12	[0.05, 0.19]
Autonomy	Professional competence	0.10	[0.04, 0.17]
Impact	Course satisfaction	0.03	[0.00, 0.07]
Impact	Professional competence	0.03	[0.00, 0.06]

Each dimension was tested separately as the mediator. Due to collinearity among the dimensions, the effect sizes should not be summed directly.

The complete indirect effect—where the direct paths from group to outcomes became non-significant after entering psychological empowerment—suggests that the reform’s statistical association operates almost entirely through students’ internalized psychological states. According to self-determination theory (Ryan and Deci, 2000), practical tasks that afford autonomy and meaning serve as “need-supportive contexts.” When students perceive their learning activities as personally meaningful (meaning) and self-directed (autonomy), they internalize external regulations, leading to deeper engagement and higher competence. The finding that meaning and autonomy contributed more than competence and impact is consistent with prior meta-analytic evidence (Seibert et al., 2011) showing that meaning is the strongest proximal predictor of intrinsic motivation in educational settings. Thus, AI-era evaluation reforms should prioritize authentic tasks that enhance meaning and provide students with real choices to foster autonomy.

### 3.3 Moderating effect of humble mentor leadership (within group C, H3)

This section tests H3,limited to within Group C (*n* = 52). With T2 psychological empowerment as the dependent variable, “intensity of intervention in Group C” (operationalized as students’ self-reported depth of engagement with practical tasks, standardized) as the independent variable, perceived humble mentor leadership as the moderator, and controlling for T1 psychological empowerment and other covariates, regression analysis showed a significant interaction between humble leadership and intervention intensity on psychological empowerment (*β* = 0.24, *p* < 0.001). Simple slope analysis (see Table 4) indicated that under high humble leadership (M + 1SD), the association between task engagement depth and psychological empowerment was positive (β_simple = 0.63, *p* < 0.001); under low humble leadership (M−1SD), this effect was weaker and only marginally significant (β_simple = 0.19, *p* = 0.058).

**Table 4:** Simple slope analysis (within Group C).

Level of humble leadership	*β*	*SE*	*t*	*p*
High (M+1SD)	0.63	0.10	6.30	<0.001
Low (M−1SD)	0.19	0.10	1.90	0.058

The moderated mediation was further tested using PROCESS macro (Model 7, 5,000 bootstrap resamples). The index of moderated mediation was 0.09 (95% CI [0.03, 0.17]), not containing zero, indicating that humble leadership substantially moderated the indirect path from “intervention intensity → psychological empowerment → learning outcomes” (Hayes, 2015). Under high humble leadership, the conditional indirect effect was 0.31 (95% CI [0.18, 0.45]); under low humble leadership, it was 0.09 (95% CI [−0.02, 0.21], containing zero). Thus, H3 was supported.

As a control, we repeated the same analysis within Groups B and A (using within-group task engagement as the independent variable). No significant moderating effects were found (*p* > 0.10). This further supports that the catalytic effect of humble leadership emerged only in the high-uncertainty, high-interaction practical task context (Group C).

**Figure 2:**
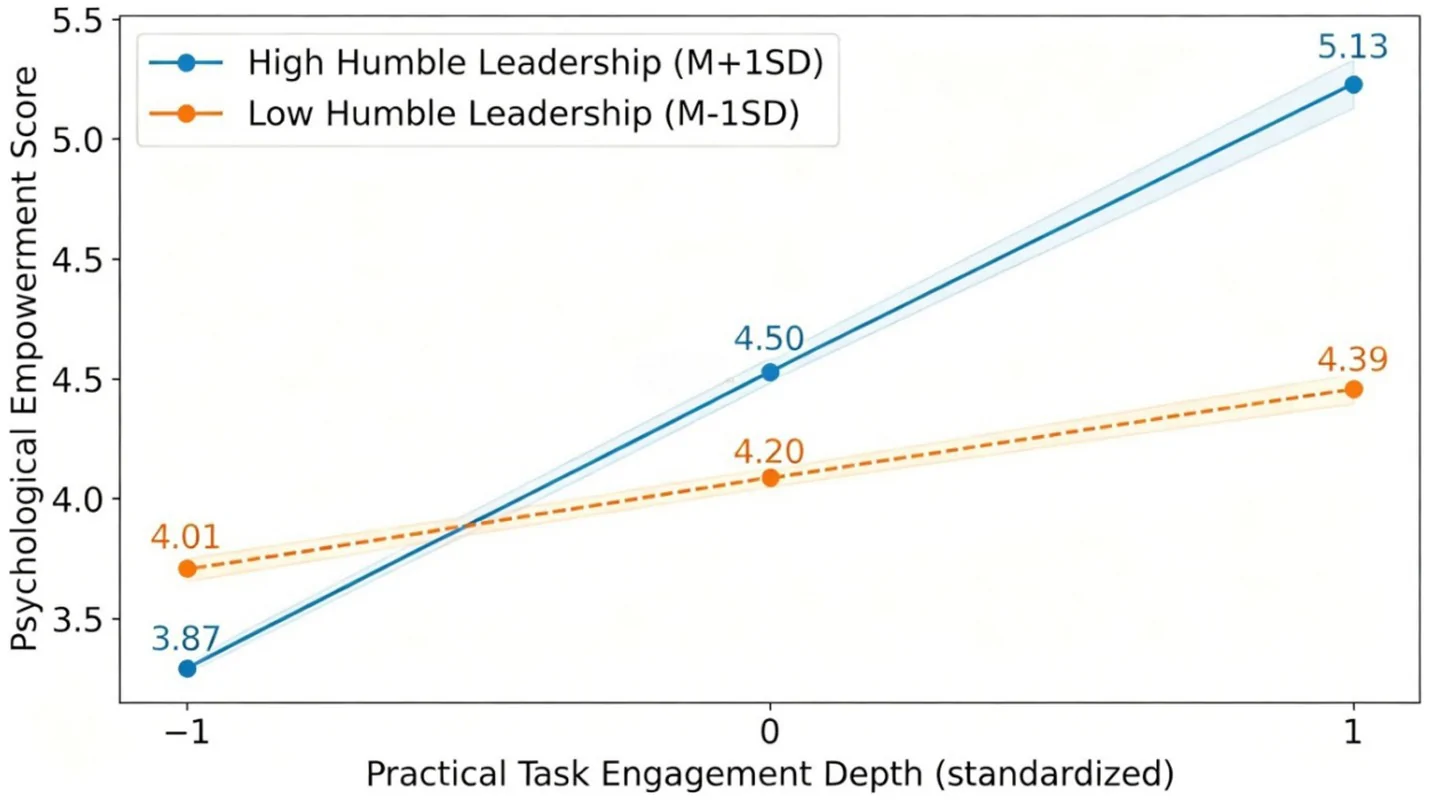
Simple slopes depicting the moderating correlational association between task engagement depth and psychological empowerment at high and low levels of perceived humble mentor leadership (within Group C only).

Figure 2 presents the simple slopes plot of this moderating effect. As shown in the figure, the positive relationship between practical task engagement depth and psychological empowerment is steeper under high humble leadership than under low humble leadership.

The significant interaction between humble leadership and intervention intensity (*β* = 0.24, *p* < 0.001) indicates that the relational context moderates the psychological internalization of reform. From the perspective of leader-member exchange theory (Graen and Uhl-Bien, 1995), high-quality teacher-student relationships—characterized by openness, mutual learning, and psychological safety—correlate with stronger student psychological engagement patterns. Under high humble leadership, supervisors’ willingness to admit limitations and learn from students signals that exploration and mistakes are tolerated, thereby encouraging students to take intellectual risks. This psychological safety (Edmondson, 1999) enables students to fully engage with practical tasks, transforming external challenges into internal growth. Under low humble leadership, the same tasks may be perceived as another form of high-stakes assessment, leading to conservative, surface-level engagement and weaker psychological empowerment.

Taken together, the mediation and moderation findings support a triple synergy framework: (a) task design (practical, authentic problems) provides the external trigger; (b) psychological empowerment (especially meaning and autonomy) serves as the internal transformation mechanism; and (c) humble mentor leadership acts as a relational catalyst that strengthens the link between task engagement and psychological empowerment. This framework moves beyond single-factor explanations (e.g., “AI training alone works”) toward a systemic view in which the observed patterns associated with AI-era evaluation reform depend on the alignment of task, person, and context. Practically, this implies that universities should not only redesign tasks and train AI skills but also cultivate humble leadership among faculty and foster psychologically safe learning environments.

### 3.4 Robustness checks: supplementary analyses

To evaluate the stability of the observed between-group patterns under the constraints of only one cluster per condition, we conducted three complementary supplementary analyses: cluster-level descriptive statistics, E-value sensitivity analysis, and permutation-based inference.

#### 3.4.1 Cluster-level descriptive patterns

We first aggregated all primary outcomes at the cluster level to assess whether the observed individual-level patterns were driven by outliers. The cluster means for the three cohorts were: Cluster A (traditional model, *n* = 52) M = 3.92 (SD = 0.81), Cluster B (AI training, *n* = 51) M = 4.35 (SD = 0.75), and Cluster C (comprehensive reform, *n* = 52) M = 4.90 (SD = 0.70) for course satisfaction; and 74.00 (8.20), 78.80 (7.00), and 86.10 (6.40) for professional competence, respectively. The rank order of cluster means (C > B > A) was consistent with the individual-level findings, suggesting that the observed patterns reflect cohort-level trends rather than isolated extreme responses.

#### 3.4.2 Sensitivity to unmeasured confounding

Following VanderWeele and Ding (2017), we calculated E-values for the primary comparisons to quantify the minimum confounding strength required to account for the observed associations. For course satisfaction (Group C vs. Group A, Cohen’s d = 1.29), the E-value was 3.6. For professional competence (Cohen’s d = 1.65), the E-value was 4.9. For psychological empowerment (Cohen’s d = 0.92), the E-value was 2.4. These values indicate that an unmeasured confounder would need to be associated with both treatment assignment and the outcomes by risk ratios of at least 2.4 to 4.9 to fully explain away the observed associations. While such magnitudes suggest that strong unmeasured confounding would be required, we cannot fully rule out latent cohort differences as alternative explanations. These findings should therefore be interpreted as suggestive rather than definitive.

#### 3.4.3 Permutation-based inference

Given the small number of clusters (*k* = 3), cluster-robust standard errors may be unreliable. To supplement the primary analyses, we conducted permutation-based inference. We computed the observed F-statistic for each primary outcome and compared it to a null distribution generated by randomly reassigning the three clusters to the three treatment conditions (all 6 possible permutations). The permutation *p*-values were 0.286 for all three outcomes. The permutation test does not deliver confirmatory inferential evidence; it merely illustrates the fundamental statistical discrimination limit of a three-cluster design, where the minimal possible permutation p-value reaches 0.143 even under large group disparities. The primary evidence for the observed patterns therefore rests on the consistency of the descriptive effects (cluster-level ranking, effect sizes, and E-values) rather than on statistical significance per se. All descriptive, sensitivity, and permutation supplementary results jointly qualify all correlational interpretations presented throughout the Results and Discussion sections.

## 4 Discussion

Before discussing the specific findings, it is essential to reiterate the exploratory nature of this study. Due to the cluster-assigned quasi-experimental design with one cluster per condition, the results reported below should be interpreted as preliminary and suggestive rather than as conclusive evidence of causal effects. The primary contribution of this study is to generate hypotheses about the potential roles of psychological empowerment and humble leadership in AI-era educational reform, which warrant further testing in more rigorous designs (e.g., multi-cluster or individually randomized trials).

### 4.1 Psychological empowerment as a statistical bridge from external reform to internal growth

One core finding is that psychological empowerment showed a statistical indirect association between the reform and learning outcomes, with meaning and autonomy contributing the most. This suggests that whether AI skills training alone (Group B) or skills training combined with practical tasks (Group C) was implemented, the statistical pathway through which these external interventions ultimately correlated with higher student satisfaction and competence was via the transmission of internal psychological states such as meaning and autonomy (Ryan and Deci, 2000; Zhang and Bartol, 2010). The contrast between Group B and Group C is particularly informative: Group B students mastered AI tools but used them within the traditional individual paper framework, which sometimes exacerbated anxiety about paper novelty or concerns about plagiarism, with limited improvement in meaning and impact. In contrast, the practical task design in Group C – solving real educational problems, choosing one’s own topics, designing research proposals – systematically incorporated the four elements of psychological empowerment, especially meaning and autonomy. This finding points to a clear psychological focus for AI-era educational evaluation reform: meaning should be strengthened through authentic tasks, and autonomy should be ensured through empowering management, so that AI can be transformed from an external “threat-substitute” or “efficiency tool” into an empowering partner for students’ self-realization (Liu and Zhang, 2024).

Nevertheless, it must be acknowledged that the cross-sectional design of this study cannot establish temporal precedence from M to Y. Future research should adopt longitudinal designs (e.g., T1, T2, T3 multiple measurements) or experimental manipulation of the mediator to more rigorously test causal direction.

### 4.2 Conditional interaction of humble leadership: a within-treatment pattern in high-uncertainty tasks

Another important finding is that the moderating effect of perceived humble mentor leadership appeared as a within-treatment interaction within Group C, and the effect tended to be weaker under low perceived humble leadership. This result aligns with the core premise of Leader-Member Exchange theory: high-quality teacher-student relationships are a key context for correlational patterns (Graen and Uhl-Bien, 1995; Hu et al., 2022). The practical tasks in Group C were characterized by novelty, exploration, and collaboration – students needed to try unfamiliar AI functions, propose possibly immature ideas, and debate in groups and public settings. In this context, the supervisor’s behavioral pattern became a critical environmental signal. Supervisors with high humble leadership – by acknowledging their own limitations in AI use, sincerely praising students’ ideas, and inviting students to explore together – essentially created a “psychologically safe learning community” (Duan et al., 2024; Edmondson, 1999). In such a community, students dared to take risks, were willing to share, and were not afraid to make mistakes, thereby gaining the greatest psychological nourishment from challenging tasks. Conversely, under low humble leadership, students tended to view the complex practice as “another exam,” behaved more conservatively, and psychological empowerment was less likely to emerge. Nevertheless, the cross-sectional and self-reported nature of the data within Group C means that the observed interaction could be influenced by reverse causation or common method variance. Consequently, the term “catalyst” is used here as a conceptual heuristic to describe the statistical interaction pattern, rather than implying a strong causal claim.

The absence of a moderating effect in Groups B and A provides counter-evidence supporting this logic: traditional paper assignments impose fewer demands on teacher-student interaction patterns, the process is relatively fixed, students need little autonomous exploration or risk-taking, and thus they are less sensitive to the relational context. This suggests that task characteristics determine the importance of the relational context. High-interaction, high-uncertainty tasks require humble mentor leadership more strongly as a “catalyst.” This finding extends the boundary conditions of leadership research from “general context” to the specific dimension of “task uncertainty.”

It should be noted, however, that because Group C supervisors received an additional humble leadership practice manual, the moderating effect observed within Group C may not be fully generalizable to naturally occurring humble leadership. This effect may partly reflect a combination of experimental induction and natural variation. Future research should measure naturally occurring humble leadership across all groups (without any manual intervention) or adopt a 2 × 2 factorial design (practical task present/absent × humble leadership training present/absent) to more cleanly separate the contributions of the two sources.

A further conceptual qualification is warranted regarding the nature of the moderator. Because humble leadership was experimentally induced in Group C (via the practice manual) rather than measured as a naturally occurring individual difference across all groups, the observed moderating effect does not support a claim about humble leadership as an independent contextual moderator. Instead, it should be interpreted as a within-treatment interaction between individual engagement depth and perceived supervisor behavior under a reform condition that included humble leadership training. This distinction is critical: the design does not permit conclusions about whether naturally occurring humble leadership would yield similar correlational patterns. Future research should adopt a 2 × 2 factorial design (practical task present/absent × humble leadership training present/absent) with measurement of baseline humility to separate the two sources of variation.

### 4.3 Triple synergy: an integrative framework and practical implications

Integrating the above findings, the observed patterns associated with AI-era evaluation reform can be conceptualized as a process synergistically driven by three elements:task design, psychological empowerment, and relational context. Task design (practical, AI-resistant) constitutes the external vehicle, establishing foundational contextual conditions for the reform. Psychological empowerment, especially meaning and autonomy, acts as the internal transformation mechanism, determining whether the trigger conditions are internalized by students and converted into deep learning motivation. Humble mentor leadership provides relational assurance, creating a psychologically safe learning environment that facilitates the transformation. This “triple synergy” framework moves beyond single-variable-driven path dependencies toward a multi-interactive systemic perspective.

The findings have three implications for current AI-empowered teaching reform in higher education.

First, curriculum evaluation reform cannot stop at “changing tasks”; it must also “change the logic” simultaneously. If task conversion is not accompanied by the embedding of psychological empowerment elements (such as meaning and autonomy), students may still approach the work perfunctorily with a “complete the required actions” mindset. The practical tasks in Group C were associated with higher observed scores not only because they adopted a group project format, but also because the evaluation weights were tilted toward competence indicators such as problem definition, critical AI use, and presentation persuasiveness, and because students had substantial autonomy in selecting topics and designing plans. In other words, the “AI-resistance” of the task format is a necessary but not sufficient condition; shifting the evaluation logic from “outcome-oriented” to “process- and competence-oriented” is the key.

Second, faculty development cannot stop at “teaching technology”; it must also “change relationships.” Most current AI training for faculty focuses on tool usage – how to use ChatGPT for lesson preparation, generate exam questions, detect AI cheating, etc. Although such training is necessary, in the AI era the role of the supervisor is shifting from “knowledge authority” to “learning partner” (Ding et al., 2022; Zhong et al., 2023). If supervisors maintain a traditional authoritative style, even when the curriculum adopts practical task reforms, students may be afraid to explore fully due to fear of making mistakes or being criticized, thereby suppressing the emergence of psychological empowerment. Therefore, faculty development should expand to include humble leadership training modules, covering how to openly admit one’s own AI limitations, how to appreciate and adopt students’ novel technical solutions, and how to proactively learn from students. Experiential training methods such as case discussions, role-playing, and behavioral checklists (like the Humble Leadership Practice Manual used in this study) can be adopted.

Third, institutional reform cannot stop at “issuing documents”; it must also “adjust the ecosystem.” The triple synergy framework reveals that reform requires coordinated progress on task, psychological, and relational levels. The absence of any one level may weaken the reform’s observed patterns. Universities could establish “AI + practice + empowerment” pilot zones for evaluation reform in selected departments, simultaneously providing humble leadership training for supervisors and psychological guidance for students, thereby forming a replicable and scalable systemic solution.

### 4.4 Limitations and future directions

Several limitations should be noted when interpreting the results.

First, and most fundamentally, the cluster-assigned quasi-experimental design with only one cluster per condition severely limits causal inference. Because each treatment condition was represented by a single cohort, the intervention is completely confounded with cohort membership. Observed differences between Groups A, B, and C may reflect not only the interventions but also pre-existing cohort characteristics, intake-year effects, institutional context, supervisor characteristics, or other unmeasured cluster-level factors. With only one cluster per condition, there is no empirical basis for disentangling treatment effects from cohort effects. Therefore, all between-group differences should be interpreted as exploratory associations rather than evidence that the intervention itself produced the observed outcomes. Future research should employ multiple clusters within each condition (or individual-level randomisation where feasible) to permit valid estimation of intervention effects.

Second, the statistical treatment of clustering is inherently limited by the extremely small number of clusters (*k* = 3). While we employed cluster-robust standard errors, this approach relies on asymptotic assumptions that are unlikely to be met with only three clusters. The resulting standard errors, *p*-values, and confidence intervals should therefore be interpreted with caution and treated as descriptive rather than inferential. More fundamentally, the effective sample size for testing treatment effects is the number of clusters (*k* = 3) rather than the number of individual participants (*N* = 155), meaning that statistical significance testing of between-group differences is not well supported. To supplement the primary analyses, we conducted permutation-based tests. However, with only three clusters, the number of possible permutations is inherently limited to 3! = 6, meaning that even the most extreme observed difference yields a permutation *p*-value of only 0.143. This further underscores that the primary contribution of this study is descriptive and hypothesis-generating rather than confirmatory. The reported findings are best viewed as generating hypotheses for future research, and future studies should include a larger number of clusters to permit reliable multilevel modeling and valid hypothesis testing.

Third, Covariates such as perceived task difficulty and individual AI training engagement were not collected in the original survey dataset; therefore, nested covariate-adjusted regression for Group C cannot be conducted. Consequently, Group C is effectively a bundled intervention consisting of practical task reform, revised evaluation procedures, and humble leadership induction. Because these components were introduced simultaneously, the study cannot determine whether the observed patterns are attributable to practical tasks, supervisory behavior, assessment reform, or their combination. This precludes a rigorous test of the proposed moderation pathway. Future research should independently manipulate humble supervisor training apart from practical task redesign to isolate its unique relational mechanism.

Fourth, the assessment of professional competence was not based on equivalent tasks across conditions. Groups A and B submitted individual course papers, while Group C submitted project-based reflective reports. These represent different forms of academic output, making direct comparisons of competence scores problematic. Although external evaluators were blinded to group assignment, the task format differences may have influenced ratings because the products reflected different skills and opportunities for demonstrating competence. Furthermore, the rubric included dimensions such as “technology integration” and “critical reflection,” which align closely with the intervention objectives. This creates a degree of circularity between the intervention design and the assessment criteria: the outcome measure may partially capture successful engagement with the intervention itself rather than underlying professional competence. While we attempted to design the rubric to focus on general academic competencies, this limitation remains. Consequently, the observed higher scores of Group C should be interpreted with particular caution. Future research should employ a common assessment task and rubric across all conditions to ensure comparability and avoid criterion contamination. This limitation is inherent to the original design and cannot be resolved *post-hoc* with the available data. Accordingly, the competence-related findings should be treated as descriptive group-level differences rather than as evidence of superior learning or skill acquisition.

Fifth, the sample was restricted to doctoral students in education. The disciplinary characteristics—emphasis on practical reflection and meaning construction—may have amplified the effects of psychological empowerment. Future research should test the cross-situational stability of the model across different disciplines such as science, engineering, medicine, and humanities.

Sixth, the intervention lasted only one semester, and the long-term dynamic evolution of psychological empowerment and humble leadership could not be examined. Follow-up longitudinal designs could examine their long-term predictive effects on graduates’ career development.

Seventh, the cross-sectional nature of the mediation analysis means that psychological empowerment and learning outcomes were measured at the same post-test time point, so temporal precedence cannot be established. The “indirect effects” reported are statistical associations and should not be interpreted as causal mediation. Future research should adopt longitudinal designs (e.g., multiple measurements over time) or experimental manipulation of psychological empowerment to test causal direction.

## 5 Conclusion

Taken together, this study provides exploratory evidence that the synergy of task redesign, psychological empowerment, and relational factors may be associated with positive student outcomes. However, the design constraints preclude causal or confirmatory interpretations. The most critical limitation concerns the professional competence assessment, which is addressed in detail below. Among all limitations, the professional competence comparison requires the most careful interpretation. The assessment tasks were not equivalent across conditions, and the scoring rubric included technology-focused dimensions that overlapped with the intervention content. Consequently, this study cannot distinguish whether the higher scores observed in Group C reflect broader competence gains or task-assessment alignment. No *post-hoc* analyses can resolve this limitation with the available data. Readers should therefore interpret the competence-related findings as purely descriptive group-level differences rather than as evidence of learning or skill acquisition. Future research must employ identical assessment tasks across conditions to permit valid competence comparisons.

## Data Availability

The original contributions presented in the study are included in the article/Supplementary material, further inquiries can be directed to the corresponding author.
