# Supplementary material for: Psychological empowerment and relational catalysis: a quasi-experimental study on graduate education evaluation reform in the AI era
Source: Front Psychol. 2026 Aug 12;17:1897845. doi: 10.3389/fpsyg.2026.1897845 (PMC13506429; doi:10.3389/fpsyg.2026.1897845)
Supplement: Supplementary file 1 [file Supplementary_file_1.pdf]

Supplementary Materials

Supplementary Table S1 to S3 for: Psychological Empowerment and Relational Catalysis: A Quasi-Experimental Study on Graduate Education Evaluation Reform in the AI Era

Manuscript ID: 1897845

Journal: Frontiers in Psychology

Supplementary Table S1

Cluster-Level Aggregated Descriptive Statistics for All Primary Outcomes

Outcome Variable	Cluster A (Traditional Model, n=52) M ± SD	Cluster B (AI Training, n=51) M ± SD	Cluster C (Comprehensive Reform, n = 52) M ± SD
Course Satisfaction	3.92 ± 0.81	4.35 ± 0.75	4.90 ± 0.70
Professional Competence	74.00 ± 8.20	78.80 ± 7.00	86.10 ± 6.40
Psychological Empowerment	4.04 ± 0.80	4.27 ± 0.71	4.72 ± 0.67
Meaning (subdimension)	4.00 ± 0.84	4.34 ± 0.78	4.95 ± 0.72
Autonomy (subdimension)	3.97 ± 0.85	4.30 ± 0.79	4.88 ± 0.73
Impact (subdimension)	4.05 ± 0.83	4.20 ± 0.77	4.45 ± 0.71
Competence (subdimension)	4.10 ± 0.82	4.25 ± 0.76	4.65 ± 0.69

Note: M = mean; SD = standard deviation. Cluster A = traditional thesis model; Cluster B = AI training only; Cluster C = comprehensive reform (AI training +

practical task-oriented practical evaluation). Consistent C > B > A ranking across all outcomes confirms cohort-level pattern coherence.

Supplementary Table S2

E-Values for All Pairwise Group Comparisons (C vs A; C vs B)

Outcome Variable	Contrast	Cohen's d	E-value
Course Satisfaction	C vs A	1.29	3.6
Professional Competence	C vs A	1.65	4.9
Psychological Empowerment	C vs A	0.92	2.4
Course Satisfaction	C vs B	0.76	2.2
Professional Competence	C vs B	1.08	2.9
Psychological Empowerment	C vs B	0.47	1.6

Note: E-values calculated following VanderWeele and Ding (2017). The E-value represents the minimum strength of association (on the risk ratio scale) that an unmeasured confounder would need to have with both treatment assignment and the outcome to fully explain away the observed association. Higher E-values indicate greater robustness to potential unmeasured confounding. All Cohen's d values are positive, indicating higher scores in Group C compared to both Group A and Group B across all outcomes.

Supplementary Table S3

Full Output of Permutation-Based Inference (Cluster-Level Randomization Test)

Outcome Variable	Observed F-statistic	All Permuted F Range	Permutation p-value
Course Satisfaction	36.52	11.21 – 36.52	0.286
Professional	54.38	18.74 – 54.38	0.286

**Outcome Variable**	**Observed F-statistic**	**All Permuted F Range**	**Permutation p-value**
Competence			
Psychological Empowerment	25.61	9.66 – 25.61	0.286

Note: The permutation test is based on all 6 possible random reassignments of the three cohorts (clusters) to the three treatment conditions (3! = 6 unique permutations). Observed F-statistic from the one-way ANOVA for each outcome. The permuted F range indicates the minimum and maximum F-values obtained across all 6 permutations. Permutation p-value = (number of permuted F-statistics ≥ observed F + 1) / (total permutations + 1). The permutation p-values are uniformly conservative due to the inherently limited permutation space (k = 3 clusters per condition), which fundamentally constrains the statistical discrimination power of this approach. These results illustrate the design limitation rather than providing confirmatory inferential evidence.
